# Inverted Patterns of Schistosomiasis and Fascioliasis and Risk Factors Among Humans and Livestock in Northern Tanzania

**DOI:** 10.3390/pathogens14010087

**Published:** 2025-01-17

**Authors:** Ephrasia A. Hugho, Yakob P. Nagagi, Lucille J. Lyaruu, Victor V. Mosha, Ndealilia Senyael, Magweiga M. Mwita, Ruth W. Mabahi, Violet M. Temba, Mapulish Hebel, Mohamed Nyati, Blandina T. Mmbaga, Theonest O. Ndyetabura, AbdulHamid S. Lukambagire

**Affiliations:** 1 Kilimanjaro Clinical Research Institute, Moshi 25102, Tanzanialukhamid@gmail.com (A.S.L.); 2 Institute of Public Health, Kilimanjaro Christian Medical University College, Moshi 25102, Tanzania; 3 Tanzania Plant Health and Pesticides Authority, Arusha 23210, Tanzania; petnagagi@gmail.com (Y.P.N.);; 4 Neglected Tropical Disease Control Program, Ministry of Health, Dodoma 40478, Tanzania; 5 Kilimanjaro Christian Medical Center, Moshi 25102, Tanzania

**Keywords:** fascioliasis, human, livestock, one health, schistosomiasis, Tanzania

## Abstract

Fascioliasis and schistosomiasis are parasitic trematodiases of public health and economic concern in humans and livestock. However, data on the distribution and risk factors for fascioliasis remain limited, while epidemiological gaps hinder schistosomiasis control in Tanzania. This One Health, cross-sectional study examined the prevalence and risk factors of schistomiasis and fascioliasis in northern Tanzania, involving 310 livestock and 317 human participants from Arusha, Kilimanjaro, and Manyara regions. Using standard parasitological methods, livestock fascioliasis prevalence was 21.3%, while schistosomiasis prevalence was 1.0%. Human fascioliasis prevalence was 1.9%, while schistosomiasis prevalence was 12.6%. Female animals, particularly cattle in Kilimanjaro and Manyara, had higher odds of fascioliasis. Human–animal contact through husbandry increased schistosomiasis risk (aOR = 4.21; 95% CI: 1.81–9.80), while the use of borehole-water was protective (aOR = 0.33; 95% CI: 0.11–0.97). Fascioliasis risk was higher among individuals aged 36–55 years (aOR = 7.66; 95% CI: 1.36–43.23), with cabbage consumption offering protection (aOR = 0.08; 95% CI: 0.01–0.89). The study revealed inverted prevalence patterns of fascioliasis and schistosomiasis in humans and livestock, driven by vector-dependent transmission dynamics. These findings emphasize the need for an integrated One Health approach to manage shared human and animal health risks in Tanzania.

## 1. Introduction

Fascioliasis and schistosomiasis are parasitic infections caused by trematodes that utilize freshwater snails as the intermediate hosts in their life cycle. Fascioliasis is mainly caused by *Fasciola hepatica* and *Fasciola gigantica* [1], while schistosomiasis is caused by *Schistosoma haematobium*, *Schistosoma mansoni*, and *Schistosoma japonicum* [2]. Infection from these parasites causes significant public health and veterinary concern, with substantial impacts on human health, livestock productivity, and the economic well-being of affected communities [3,4,5].

It is estimated that fascioliasis affects about 2.4 million people each year from 70 countries globally [6]. Fascioliasis is classically regarded as a zoonotic infection predominantly affecting livestock but is increasingly recognized as a human health threat, particularly in endemic regions [7]. Recently, a significant rise in the fascioliasis prevalence in humans and livestock has been observed [8,9]. Likewise, schistosomiasis causes over 200,000 deaths in Sub-Saharan Africa (SSA) every year, with more debilitating effects in children [10]. 

Emerging evidence highlights complex epidemiological patterns of these infections, including distinct spatial and host-related distributions [3]. In many endemic areas, schistosomiasis is more prevalent in humans than livestock hosts [2,11], while fascioliasis affects livestock more compared to humans [12,13]. This inverted pattern, however, may vary across geographical settings due to environmental, behavioral, and the intermediate freshwater snail-specific factors influencing transmission dynamics [1,14,15,16]. A combination of environmental and socio-behavioral factors appears to drive the transmission of fascioliasis and schistosomiasis, with water-use behavior, grazing practices, and regional disparities in snail habitats being the point of focus in One Health approaches for integrated control strategies [17,18,19,20,21,22]. These factors are crucial for developing sustainable interventions, particularly given the One Health implications of these diseases in shared human and animal environments.

In Tanzania, significant efforts have been made to combat human schistosomiasis, achieving high intervention coverage among endemic and high-risk populations [23]. In contrast, human fascioliasis receives comparatively little attention, resulting in limited data on its disease burden due to underreporting and low research prioritization [24,25]. Despite this lack of attention, the disease significantly impacts the economy, resulting from liver condemnations and reduced carcass weights in slaughtered livestock [5,26,27]. Studies designed around multi-disciplinary, One Health investigations can add much needed knowledge and fill gaps around the epidemiology and transmission dynamics of the trematodiases, especially where active monitoring across all host species may be difficult to achieve. Integrated and inter-operable animal, human, and environmental health systems can also raise the alert level of possible outbreaks or environmental drivers of infection to facilitate early detection and response to outbreaks of such interlinked zoonotic diseases [25,28,29]. Given the shared environments and interconnected health of humans, animals, and ecosystems, a One Health approach is essential to address these challenges. This integrated perspective can enhance the understanding of disease dynamics, improve surveillance, and inform interventions targeting both human and animal health. Accordingly, this study aimed to assess the prevalence and risk factors of schistosomiasis and fascioliasis infection among humans and livestock in northern Tanzania, emphasizing the importance of One Health in mitigating the burden of these zoonotic diseases.

## 2. Methodology

### 2.1. Study Design, Settings, and Population

**Figure 1:**
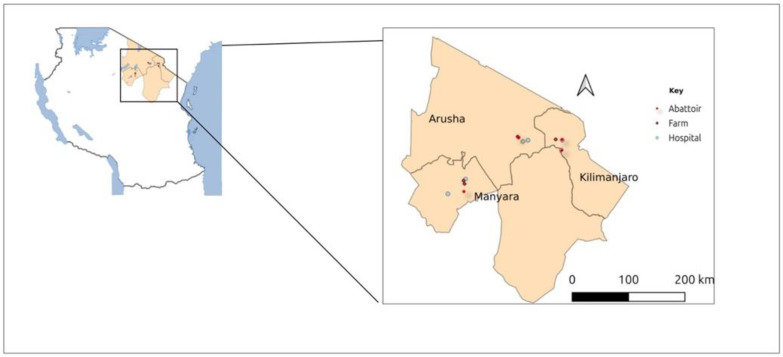
Map of Tanzania showing sampling regions.

A cross-sectional study was conducted in three regions of the northern zone of Tanzania, namely, Kilimanjaro, Arusha, and Manyara, from November 2023 to August 2024 (Figure 1). Two health facilities were selected from each region to recruit study participants. In the Kilimanjaro region, Tanganyika Planting Company (TPC) Hospital and Pasua Health Center were chosen; in the Arusha region, Total Care Health Center and Meru Hospital were selected; and in the Manyara region, Dareda Mission Hospital and Magugu Health Center were considered. Caretakers of children and adult patients visiting the healthcare facilities with symptoms of trematode infection (such as recurring fevers, abdominal discomfort, bloody diarrhea, urinary complications such as haematuria, rash, and allergic skin reactions, hepatomegaly, with/without ascites, and jaundice [24,30]) were approached for consent and inclusion in the study. Participants living in the study area were also included to allow for risk factor analysis. Participants who were unable or unwilling to consent, and those who reported having taken any anthelmintic medications within the past two weeks were excluded to avoid false negativity of stool analysis. Livestock sampling from these regions was performed in abattoirs, slaughter houses, and farms near the selected health facilities.

### 2.2. Sample Size and Sampling

The previous health facility-based prevalence of fascioliasis (21%) was used to determine the sample size [24]. A minimum of 300 participants in three regions was obtained to estimate the local prevalence with a 95% CI and ≤5% margin of error. For animal sampling, approximately 100 livestock fecal samples (50 fecal samples from farm animals and 50 from intestinal contents immediately collected after animals were slaughtered at abattoirs) in each region were collected for this survey. Convenient sampling was used for both humans and animals at abattoirs or slaughterhouses, whereas for farm animal selection, simple random sampling was used.

### 2.3. Sample Collection

Fresh stool (~10 g) and urine (20 mL) samples were collected from each enrolled participant. Samples were collected by the participants themselves (or with the help of a parent/guardian for minors), following instructions for sample collection provided by the study personnel. All samples were kept in appropriate leak-proof primary containers and transported chilled to the Kilimanjaro Clinical Research Institute–Biotechnology laboratory in Moshi, on the day of sampling. For livestock sampling, fresh droppings from farm animals were collected from cattle, goats, and sheep from six farms, and intestinal contents/fecal samples from the slaughtered animals were collected from 4 abattoirs. The inspection reports on the liver and mesenteric vein were recorded at abattoirs.

### 2.4. Laboratory Procedures

Wet preparation was initially performed on each human stool sample collected for examination of *S. mansoni* and *Fasciola* eggs, followed by the Kato–Katz technique as a confirmatory test following standard procedures established previously [31]. Animal fecal samples were analyzed by the filtration method established for the parasitological detection of trematode eggs in ruminants [32]. Briefly, approximately 3 g of feces was homogenized in 50 mL of normal saline and mixed thoroughly using a tongue blade. The fecal suspension was filtered using a strainer, first at 180 μm, followed by 35 μm with tap water. The filtrate was discarded and the material at the bottom of the 35 μm strainer backwashed, collected, and left to sediment for 30 min. The supernatant was decanted and the sediment re-suspended in distilled water for another 30 min. This procedure was repeated until the supernatant became clear. Next, the supernatant was discarded very carefully. The resulting sediment was stained with 1% methylene blue (Loba Chemie PVT. Ltd., Mumbai, India) (to stain the background material) and then transferred to a micro slide and covered with a coverslip, ready for examination under a light microscope at 10x magnification.

For urine analysis, a microscopic analysis of urine sediment was performed to examine the presence of *S. haematobium* eggs. Briefly, the specimen was immediately centrifuged at 3000 rpm for 3 min. The supernatant fluid was discarded, and the sediment was transferred to a slide, covered with glass, and examined using the 10x objective. The presence/absence of parasitic eggs was reported for every specimen.

### 2.5. Questionnaire Data Collection

A structured, closed-ended questionnaire was administered to all enrolled participants by the trained study personnel. The questionnaire was created based on the literature on *Fasciola* and *Schistosoma* infections, and the WHO’s recommendations, to make sure that it addressed the most pertinent information. A small group of five volunteers participated in a pre-testing of the tool to evaluate the uniformity and clarity of the responses. The questionnaire was improved by taking into account the input from this process. Some questions were reworded for greater clarity, more response options were added, and unnecessary or redundant questions were eliminated following the volunteers’ suggestions. The completed questionnaire was translated into Swahili and administered to each participant (and the parents or guardians for children) by a pre-trained member of the study team. An electronic database (Research Electronic Data Capture, REDCap; Vanderbilt University, Nashville) was used to capture responses. For livestock, the farm owners were asked to provide the information on the age of the animals sampled, status including variables such as pregnancy and lactation, age category (adult or juvenile), and deworming history. Livestock officers at the abattoir/slaughterhouses were asked to report on the liver and mesenteric vein inspection. This information was recorded on an Excel sheet.

### 2.6. Data Analysis

STATA version 15 (STATA Corp, College Station, TX, USA) was used for data analysis. For the human dataset, socio-demographic characteristics were summarized by frequency and percentages, and measures of central tendency and dispersion where appropriate. A Chi-squared test was performed to determine the relationship between *Fasciola* or *Schistosoma* infection status and explanatory variables. Bivariate and multivariable logistic regression was performed to determine the association between explanatory variables and the presence of parasitic eggs under investigation. Variable selection for the final model was based on a *p*-value of less or equal to 0.2 in bivariate analysis to overcome important confounders. A *p*-value of less than 0.05 was considered statistically significant. The same approach was applied to the livestock dataset.

## 3. Results

### 3.1. Livestock and Human Demographics

In this study, a total of 627 samples were analyzed, with 310 fecal samples from livestock and 317 stools samples from human participants. Animal fecal samples were collected from cattle (*n* = 252), goats (*n* = 39), and sheep (*n* = 19). These samples were collected from both live animals (*n* = 152) and those in slaughterhouses (*n* = 158). The distribution of samples across Arusha, Kilimanjaro, and Manyara was 102, 107, and 101, respectively (Table 1).

Among the 317 human participants, 103 were from Arusha, 114 from Kilimanjaro, and 100 from Manyara. Their ages ranged from 1 to 72 years, with a median age of 28 years (IQR 17–42), and a slight female predominance (57.1%; *n* = 181/317). The majority had at least primary education (89.9%) (Table 2)

### 3.2. Prevalence of Fascioliasis and Schistosomiasis Infections

**Figure 2:**
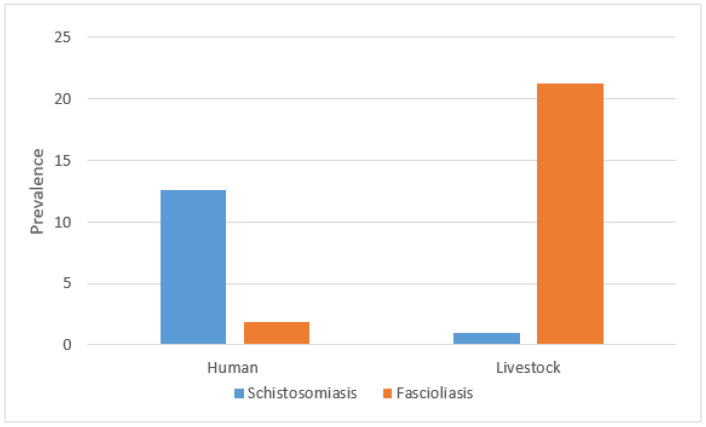
Prevalence of schistosomiasis and fascioliasis in humans and livestock.

Overall, the prevalence of fascioliasis in livestock across all regions was 21.3% (95% CI: 16.7–25.8%), while schistosomiasis prevalence was lower at 1.0% (95% CI: −0.1–2.1%). In humans, the prevalence of fascioliasis was 1.9% (95% CI: 0.4–3.4%) and schistosomiasis prevalence was 12.6% (95% CI: 8.9–16.3%). The prevalence of co-infection was 0.3% for both humans (*n* = 1/317) and livestock (*n* = 1/310) (Figure 2).

### 3.3. Prevalence of Fascioliasis and Schistosomiasis Infections by Region

**Figure 3:**
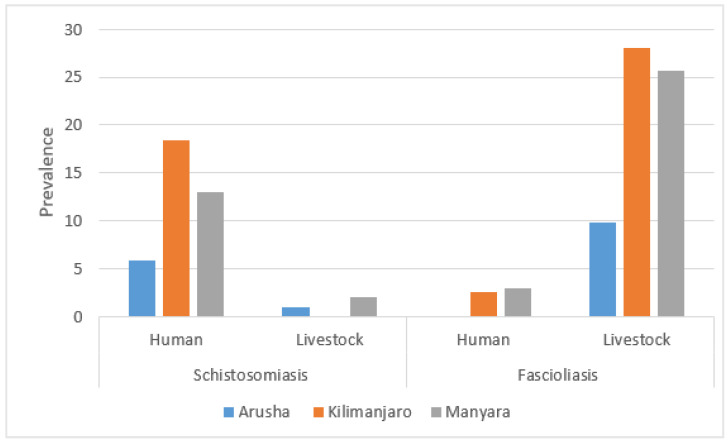
Prevalence of schistosomiasis and fascioliasis by sampling population and region.

The prevalence of fascioliasis among livestock in Arusha was 9.8%, while in Kilimanjaro and Manyara it was 28.0% and 25.7%, respectively. Schistosomiasis prevalence among livestock showed a different regional variation, with Arusha at 1.0% (*n* = 1/102), Kilimanjaro at 0% (*n* = 0/107), and Manyara at 2.0% (*n* = 2/101). In human samples, the overall prevalence of fascioliasis was 1.9% (*n* = 6/317), and schistosomiasis prevalence was 12.6% (*n* = 40/317), with 1 case of schistosomiasis identified from urine and the remaining 39 cases from stool samples. The prevalence of fascioliasis was 0% among participants from Arusha, 2.6% from Kilimanjaro, and 3.0% from Manyara. Schistosomiasis prevalence in humans was 5.8%, 18.4%, and 13.0% in Arusha, Kilimanjaro, and Manyara, respectively (Figure 3).

### 3.4. Prevalence of Fascioliasis and Schistosomiasis by Sample Source (Livestock Population) and Human Demographics

When examining infection rates by sample type, fascioliasis was observed in 21.7% (*n* = 33/152) of live animal samples and in 20.9% (*n* = 33/158) of slaughterhouse samples. Schistosomiasis prevalence was 1.3% in slaughterhouse samples (*n* = 2/158) and 0.7% in live animal samples (*n* = 1/152). More details are found in Table 3.

Among human participants, schistosomiasis prevalence was higher in males (15.4%) than females (10.5%) and varied across age groups, with the highest prevalence observed in those aged 36–55 years (14.8%) and <18 years (14.6%). It was most prevalent in individuals with no formal (15.6%) or primary education (16.7%) and those residing in the Kilimanjaro region (18.4%). Economic activities involving fishing (22.2%) and crop farming (15.7%) showed higher prevalence, as well as water-related behaviors like swimming (15.4%). Relying on undeveloped wells or lakes as a primary water source was found to have a schistosomiasis prevalence of 15.8% and 16.7%, respectively. Fascioliasis prevalence was higher in females (2.8%) than males (0.7%) and peaked in individuals aged 36–55 years (5.6%). Primary education (5.0%), crop farming (3.6%), and reliance on ponds (2.4%) was linked to slightly higher prevalence (Table 4).

### 3.5. Risk Factors Associated with Fascioliasis and Schistosomiasis

In livestock, the bivariate analysis showed that goats had lower odds of *Fasciola* infection than cattle (OR = 0.08; 95% CI: 0.01–0.59). Another significant association was found with higher odds of infection for animals sampled from the Kilimanjaro and Manyara regions (*p*-value < 0.05). In multivariable analysis, we found that female animals (adjusted OR = 2.17; 95% CI: 1.18–3.99) and those grazing or slaughtered in Kilimanjaro and Manyara had high odds of *Fasciola* infection. Lower odds of infection were found for goats and sheep as compared to cattle, which translates to the lower representation of these species in the population (Table 5). Risk factor analysis for schistosomiasis was not performed due to a lower number of positive cases detected.

In human participants, those from the Kilimanjaro region had higher odds of schistosomiasis than those from Arusha and Manyara (adjusted OR: 2.33; 95% CI: 1.15–4.71). In addition, involvement in animal husbandry was significantly associated with more than four times higher odds of schistosomiasis (adjusted OR = 4.21; 95% CI: 1.81–9.80), while using water from boreholes was significantly associated with lower odds of schistosomiasis infection (adjusted OR = 0.33; 95% CI: 0.11–0.97) (Table 6). For fascioliasis, individuals aged between 36 and 55 years had seven times higher odds of fascioliasis than other age groups (adjusted OR = 7.66; 95% CI: 1.36–43.23), likely due to longer exposure to contaminated environments. Significantly lower odds of infections were noted for participants who reported having consumed cabbage as the common vegetable (adjusted OR = 0.08; 95% CI: 0.01–0.89) (Table 7).

## 4. Discussion

This study was conducted to determine the prevalence of fascioliasis and schistosomiasis in humans and animals and the associated risk factors in Kilimanjaro, Arusha, and Manyara in northern Tanzania. The findings from this study demonstrate a significant burden of fascioliasis in livestock, with an overall prevalence of 21.3%, compared to a much lower prevalence of schistosomiasis (1.0%). In humans, the reverse pattern was observed, with schistosomiasis prevalence being higher (12.6%) than fascioliasis (1.9%). Risk factors for fascioliasis in livestock were animal species, sex, and sampling region. In humans, age and cabbage consumption were significant predictors of fascioliasis, whereas sampling region, animal husbandry, and water source were significant risk factors identified for schistosomiasis.

We found regional differences in infection rates, particularly for fascioliasis in livestock, where prevalence was high in Kilimanjaro (28.0%) and Manyara (25.7%) and lower in Arusha (9.8%). This pattern may be attributed to environmental factors such as the availability of snail habitats in these areas which are intermediate hosts for these trematodes, and human activities including irrigation [33,34,35]. For example, in the study areas of Kilimanjaro and Manyara regions, 34,519 and 6000 hectares, respectively, are under irrigation systems, out of the 44,190 and 15,460 hectares declared potential for irrigation [36,37]. While Tanzania benefits from regular rainfall due to its equatorial location, commercial agriculture often necessitates irrigation, particularly for cultivating crops outside their natural seasons. However, irrigation practices are not confined to specific areas but are widely adopted as needed to support agricultural activities. Cattle were found to have a high prevalence of *Fasciola* compared to goats and sheep. However, there was a skewed distribution in sampling across animal species and regions. Previous studies reported a high prevalence of fascioliasis in cattle [38,39]. The similar prevalence of fascioliasis observed in slaughterhouses and live animal samples (21.7% vs. 20.9%) suggests that the detection of infection is consistent across sample types, indicating the reliability of the study’s diagnostic methods for livestock. These results highlight the importance of fascioliasis as a major parasitic infection in livestock in Tanzania, aligning with previous reports indicating its economic impact due to liver condemnations and reduced carcass weight [5,26,27]. On the other hand, schistosomiasis prevalence in livestock was low across all regions, reflecting limited animal exposure to water sources contaminated with *Schistosoma* spp. cercariae. The slightly higher prevalence in slaughterhouse samples compared to live animals (1.3% vs. 0.7%) may reflect differences in exposure, as slaughtered animals are often procured from diverse locations, potentially including areas with higher transmission risk or lower risk too. Similar patterns have been reported in Côte d’Ivoire, with low schistosomiasis and high fascioliasis prevalence [39].

In humans, schistosomiasis prevalence was highest in Manyara (18.4%) and Kilimanjaro (13.0%), likely due to greater reliance on freshwater sources for daily activities, increasing exposure to *Schistosoma* spp. Varying prevalence of schistosomiasis has been reported in other regions in the country [40,41,42,43], indicating that schistosomiasis remains a priority due to its higher burden and recognized impact on morbidity. On the other hand, fascioliasis prevalence in humans was slightly higher in Manyara (3.0%) compared to Kilimanjaro (2.6%), with no cases detected in Arusha. A previous study in Arusha reported a fascioliasis prevalence of 21% [24]; hence, the lower prevalence of fascioliasis might result from the sampling approach in the current study being biased towards schistosomiasis over fascioliasis. Previous studies have reported poor fecal egg yield in patients presenting to health facilities with acute infection (abdominal) symptoms, as the noted period of *Fasciola* egg shedding typically takes place 8 to 13 weeks after initial infection (acute presentation) [25,44]. Overall, there is underreporting of fascioliasis in humans, attributed to a lack of prioritization in both clinical and research in developing countries including Tanzania. The existing evidence shows an increasing burden of the disease in humans, hence requiring more attention in future [8,24]. The current study benefited from a comprehensive One Health approach, allowing the investigation of human health, livestock health, and environmental factors to observe the various prevalences and risk factors for *Fasciola* and *Schistosoma* infection. This approach to study design highlights the added value of a multi-faceted, multi-disciplinary approach to a complex disease landscape. Although the individual host and spatial characteristics observed may have had limited representation, the overall ecosystem examination indeed demonstrates the complex patterns and transmission dynamics of both fascioliasis and schistosomiasis in humans and livestock. A global review of the existing research highlights the significant burden of fascioliasis in Africa [12,45,46]. This suggests that more multi-disciplinary research, community awareness and engagement, and the risk mapping of disease hotspots are needed to inform policy changes for improving diagnosis and preventing long-term consequences associated with fascioliasis in humans.

In the risk factor analysis, the higher odds of fascioliasis infection observed in livestock sampled from Kilimanjaro and Manyara as compared to Arusha suggest that these regions might present potential hotspots for fascioliasis and warrant targeted interventions. While these findings are statistically significant, they should be interpreted with caution, considering variations in species distribution across regions, and further studies with larger, more representative samples are needed to confirm these results. Regional variations in the prevalence of fascioliasis are reported in other countries [9,13,18]. We found that female animals had higher odds of infection as compared to male animals. Female animals’ increased risk could be linked to longer lifespans, directed to reproduction benefits or differing grazing behaviors that increase exposure to contaminated environments [47]. In addition, the pregnancy and lactation periods in female animals are thought to be a stressful physiological element that reduces their resistance to infections [17,27,48,49]. Further qualitative studies into the socio-economic drivers of disease transmission are still needed to develop a comprehensive, community-focused understanding of the myriad and complex risk behaviors as well as climatic and environmental factors of the zoonotic trematode infections in Tanzania.

The association of fascioliasis with older age (36–55 years) in humans likely reflects cumulative exposure over time. This is contrary to previous studies that have indicated a high risk of infection at younger ages [24]. This can be explained by the fact that our data indicate that all *Fasciola*-positive cases had primary education, which may reflect a reduced awareness of zoonotic disease transmission and preventive measures. Additionally, within the 36–55 age group, two of the three cases were engaged in crop-growing activities, likely involving direct exposure to contaminated water, thereby increasing their vulnerability. Conversely, the protective effect of consuming cabbage may indicate that this vegetable is less likely to harbor *Fasciola* metacercariae than watercress and other aquatic plants, or is less often eaten raw as compared to the latter water vegetables. Other studies have indicated increased odds of fascioliasis associated with the consumption of radish and other raw vegetables [14,19,46]. For schistosomiasis, the strong association with animal husbandry underscores the occupational risk posed by frequent exposure to freshwater environments contaminated with *Schistosoma* spp. Residing in the Kilimanjaro region was linked to a significantly higher likelihood of schistosomiasis compared to Arusha and Manyara. Recent research has highlighted a notable prevalence of schistosomiasis among both children and adults, suggesting that the active transmission of the disease persists in the area [50,51]. Using borehole water was a protective factor for schistosomiasis. Boreholes provide a protected and isolated water source, minimizing the chances of contamination by freshwater snails which thrive in surface water sources. Other studies have reported greater odds of infection associated with unimproved water sources which are prone to contamination from the infected snails [14].

## 5. Study Limitations

This study had a number of limitations that may have affected the quality and generalizability of the main findings. Firstly, we had a biased sampling approach that targeted schistosomiasis over fascioliasis presentation, including a history of anti-helminthic use, which would not be effective against *Fasciola.* Previous studies have shown a slight skew of high-risk populations to be female children of the school-going age range (9–11 years). Furthermore, sampling at-risk populations from health facilities as opposed to the community level would generally miss the peak egg-shedding period of trematode infection, which generally falls 6 to 13 weeks after patients generally visit the health facilities with gastro-intestinal symptoms of infection. The cross-sectional sampling techniques adopted around the El Niño rains did not capture the flooding effects before and after the heavy rains. Temporal and weather factors affect the density and infection of freshwater snail vectors of both trematodiases, which multiple sampling points would best capture in time and across different weather circumstances. It is speculated that floods and heavy rains, such as those caused by El Niño, may wash freshwater snails downstream from their original locations. As a result, associating the prevalence of infections with the presence of freshwater snails in nearby water bodies becomes challenging. Peak prevalence, typically observed after normal seasonal rains, may be significantly reduced due to the impact of flooding. Additionally, many more patients visit healthcare facilities during and after flooding for a wide range of seasonal issues, many of whom may not present symptoms that would typically justify stool analysis for worm infestation. A single stool sample was also collected from each participant, which may have reduced the sensitivity of microscopic diagnostic techniques used, as egg shedding typically occurs in bursts that may not coincide with acute abdominal symptoms that many participants visited the health facilities for and, therefore, are also easier to miss by conveniently collecting only a single stool sample.

Future studies could focus on community-based sampling designs, targeting typically at-risk populations, and observing for infection at multiple time points in the year typical of peak seasons of infection, as highlighted by other studies [24,44,52]. While we aimed to sample animals from the designated study areas, the movement of livestock between regions could have influenced the prevalence rates observed in our study. Future studies could benefit from stringent control over animal sourcing or the inclusion of detailed tracking of animal to minimize this confounding factors. Future studies could also assess the impact of climate change and snail vector ecology in the transmission dynamics of fascioliasis and schistosomiasis in both human and animal populations. Capitalizing on One Health approaches that maximize community and stakeholder engagement might also pave the way for further preventive and management interventions that complement the current national chemotherapeutic-based control programs for schistosomiasis [28,53], while also addressing fascioliasis through targeting the snail vectors.

## 6. Conclusions

This study found contrasting prevalence patterns and risk factors for fascioliasis and schistosomiasis in livestock and humans in Tanzania. The classic, inverted prevalence of both trematodiases in human and animal populations strongly implies the critical role of the intermediate hosts, freshwater snails, in the transmission dynamics between the different host species. While schistosomiasis continues to be a public health priority in humans, fascioliasis remains a significant concern in livestock, with important implications for both health and economic productivity. More standardized One Health methods are required to enhance the surveillance and control of fascioliasis in humans considering the interconnectedness of humans, animals, and the environment. Further, continuing education programs for communities about risk factors for the transmission of *Fasciola* and *Schistosoma* could reduce human infection rates. The findings emphasize the need for increased research focus and resource allocation for fascioliasis, which remains underreported despite its significant economic and public health implications.

## Figures and Tables

**Table 1:** Livestock characteristics (N = 310).

Variable	Region *n* (%)			Total *n* (%)	ꭓ^2^ *p*-Value
	Arusha	Kilimanjaro	Manyara		
Species					0.002
Cattle	91 (89.2)	75 (70.1)	86 (85.1)	252 (81.3)	
Goat	6 (5.9)	20 (18.7)	13 (12.9)	39 (12.6)	
Sheep	5 (4.9)	12 (11.2)	2 (2.0)	19 (6.1)	
Source					0.920
Abattoir	52 (51.0)	56 (52.3)	50 (49.5)	158 (51.0)	
Farm	50 (49.0)	51 (47.)	51 (50.5)	152 (49.0)	
Age (years)					0.000
<2.5	11 (10.8)	22 (20.6)	48 (47.5)	81 (26.1)	
2.5–8	91 (89.2)	85 (79.6)	53 (52.5)	229 (73.9)	
Sex (*n* = 302)					0.079
Male	53 (52.0)	57 (57.6)	68 (67.3)	178 (58.9)	
Female	49 (48.0)	42 (42.4)	33 (32.7)	124 (41.1)	

**Table 2:** Demographic characteristics of human participants (N = 317).

Variable	Region			Total	ꭓ^2^ *p*-Value
	Arusha	Kilimanjaro	Manyara		
Age (years)					
<18	23 (22.3)	34 (29.8)	25 (25.0)	82 (25.9)	0.089
18–35	49 (47.6)	49 (43.0)	35 (35.0)	133 (42.0)	
36–55	19 (18.4)	19 (16.7)	16 (16.0)	54 (17.0)	
Above 55	12 (11.6)	12 (10.5)	24 (24.0)	48 (15.1)	
Gender					
Female	52 (50.5)	61 (53.5)	68 (68.0)	181 (57.1)	0.026
Male	51 (49.5)	53 (49.5)	32 (32.0)	136 (42.9)	
Education level					
No formal education	11 (10.7)	7 (6.1)	14 (14.0)	32 (10.1)	0.000
Primary level	26 (25.2)	56 (49.1)	38 (38.0)	120 (37.8)	
Secondary level	35 (34.0)	44 (38.6)	42 (42.0)	121 (38.2)	
Tertiary level	31 (30.1)	7 (6.1)	6 (6.0)	44 (13.9)	
Economic activities					
Crop growing					
No	86 (83.5)	101 (88.6)	47 (47.0)	234 (73.8)	0.000
Yes	17 (16.5)	13 (11.4)	53 (53.0)	83 (26.2)	
Fishing					
No	101 (98.1)	113 (99.1)	85 (85.0)	299 (94.3)	0.000
Yes	2 (1.9)	1 (0.9)	15 (15.0)	18 (5.7)	
Fish farming					
No	101 (98.1)	113 (99.1)	97 (97.0)	311 (98.1)	0.523
Yes	2 (1.9)	1 (0.9)	3 (3.0)	6 (1.9)	
Animal husbandry					
No	84 (81.6)	98 (86.0)	84 (84.0)	266 (83.9)	0.677
Yes	19 (18.4)	16 (14.0)	16 (16.0)	51 (16.1)	
Business					
No	67 (65.0)	82 (71.9)	82 (82.0)	231 (72.9)	
Yes	36 (35.0)	32 (28.1)	18 (18.0)	86 (27.1)	0.024
Employed					
No	83 (80.6)	77 (67.5)	90 (90.0)	250 (78.9)	
Yes	20 (19.4)	37 (32.5)	10 (10.0)	67 (21.1)	0.000
Other *					
No	79 (76.7)	82 (71.9)	82 (82.0)	243 (76.7)	
Yes	24 (23.3)	32 (28.1)	18 (18.0)	74 (23.3)	0.221

* Categories with lower frequencies (children, students, unemployed, drivers, and laborers).

**Table 3:** Prevalence of *Fasciola* in livestock population (N = 310).

Variable	Cattle		Goat		Sheep	
	Total, Positive	Prevalence (95% CI)	Total, Positive	Prevalence (95% CI)	Total, Positive	Prevalence (95% CI)
**Farm Animals**						
Region						
Arusha	45, 3	6.67 (−0.62–13.95)	2, 0	0	3, 0	
Kilimanjaro	39, 16	41.03 (25.59–56.46)	9, 0	0	3, 1	33.33 (−20.01–86.68)
Manyara	43, 12	27.91 (14.50–41.31)	7, 1	14.29 (−11.64–40.21)	1, 0	
Sex						
Male	32, 4	12.50 (1.04–23.96)	7, 1	14.29 (−11.64–40.21)	2, 1	50 (−19.30–119.30)
Female	87, 24	27.59 (18.19–36.98)	0		5, 0	
Status						
Pregnant	5, 2	40.00 (−2.94–82.94)	0	0	1, 0	
Lactating	18, 5	27.78 (7.09–48.47)	2, 0	0	1, 0	
NA	104, 24	23.08 (14.98–31.17)	16, 1	6.25 (−5.61–18.11)		
Age (years)						
Below 2.5	37, 11	29.73 (15.00–44.46)	8, 1	12.5 (−10.42–35.42)	4, 1	25 (−17.44–67.44)
2.5–8	90, 20	22.22 (13.63–30.81)	10, 0	0	3, 0	
Abattoir animals						
Region						
Arusha	46, 7	15.22 (4.84–25.60)	4, 0	0	2, 0	
Kilimanjaro	36, 12	33.33 (17.93–48.73)	11, 0	0	8, 1	11.11 (−10.42–35.42)
Manyara	43, 13	30.23 (16.51–43.96)	4, 0	0	1, 0	
Sex						
Male	114, 25	21.93 (14.33–29.53)	14, 0	0	9, 0	
Female	11, 7	63.64 (35.21–92.07)	7, 0	0	3, 1	33.33 (−20.01–86.68)
Age (years)						
Below 2.5	12, 1	8.33 (7.30–23.97)	13, 0	0	7, 1	14.29 (−11.64–40.21)
2.5–8	113, 31	27.43 (19.21–35.66)	8, 0	0	5, 0	

**Table 4:** Prevalence of *Schistosoma* and *Fasciola* infections in human population (N = 317).

Variable	*Schistosoma*		*Fasciola*	
	Total, Positive	Prevalence (95% CI)	Total, Positive	Prevalence (95% CI)
Gender				
Female	181, 19	10.49 (6.03–14.96)	181, 5	2.80 (0.37–5.15)
Male	136, 21	15.44 (9.37–21.51)	136, 1	0.73 (−0.74–2.17)
Age (years)				
<18	82, 12	14.63 (6.98–22.28)	82, 1	1.22 (−1.21–3.59)
18–35	133, 14	10.53 (5.31–15.74)	133, 1	0.75 (−0.72–2.22)
36–55	54, 8	14.81 (5.34–24.29)	54, 3	5.56 (−0.55–11.66)
Above 55	48, 6	12.5 (3.14–21.86)	48, 1	2.08 (−1.96–6.12)
Education level				
No formal education	32, 5	15.63 (3.04–28.21)		
Primary level	120, 20	16.67 (9.99–23.33)	120, 6	5.00 (1.10–8.90)
Secondary level	121, 12	9.92 (4.59–15.24)		
Tertiary level	44, 3	6.82 (−0.63–14.23)		
Region				
Arusha	103, 6	5.83 (1.30–10.33)		
Kilimanjaro	114, 21	18.42 (11.30–25.54)	114, 3	2.63 (−0.31–5.57)
Manyara	100, 13	13 (6.41–19.59)	100, 3	3.00 (−0.34–6.34)
Economic activities				
Crop growing				
No	234, 27	11.54 (7.45–15.63)	234, 3	1.28 (−0.16–2.72)
Yes	83, 13	15.66 (7.84–23.48)	83, 3	3.61 (−0.40–7.63)
Fishing				
No	299, 36	12.04 (8.35–15.73)	299, 6	2.01 (0.42–3.56)
Yes	18, 4	22.22 (3.02–41.43)	18, 0	
Fish farming				
No	311, 38	12.22 (8.58–15.86)	311, 6	1.93 (0.40–3.46)
Yes	6, 2	33.33 (−4.39–71.05)		
Animal husbandry				
No	266, 27	10.15 (6.52–13.78)	266, 6	2.26 (0.40–4.04)
Yes	51, 13	25.49 (13.53–37.45)		
Activities involving water contact				
Swimming				
No	278, 34	12.23 (8.38–16.08)	278, 5	1.80 (0.24–3.36)
Yes	39, 6	15.38 (4.06–26.71)	39, 1	2.56 (−2.40–7.52)
Drinking				
No	57, 4	7.02 (0.39–13.65)	57, 1	1.75 (−1.65–5.16)
Yes	260, 36	13.85 (9.65–18.04)	260, 5	1.92 (0.25–3.59)
Irrigation				
No	235, 28	11.91 (7.77–16.06)	235, 4	1.70 (0.05–3.36)
Yes	82, 12	14.63 (6.98–22.28)	82, 2	2.44 (−0.90–5.78)
Playing				
No	297, 38	12.79 (8.99–16.59)	297, 5	1.68 (0.22–3.15)
Yes	20, 2	10 (−3.14–23.15)	20, 1	5.00 (−4.55–14.55)
Drinking animals				
No	253, 30	11.86 (7.87–15.84)	253, 4	1.58 (0.04–3.12)
Yes	64, 10	15.63 (6.73–24.52)	64, 2	3.13 (−1.14–7.39)
Bathing				
No	100, 13	13 (6.41–19.59)	100, 2	2.00 (−0.744–4.74)
Yes	217, 27	12.44 (8.05–16.83)	217, 4	1.84 (0.05–3.63)
Fishing				
No	272, 35	12.87 (8.89–16.85)	272, 5	1.84 (0.24–3.43)
Yes	45, 5	11.11 (1.93–20.29)	45, 1	2.22 (−2.08–6.53)
Common sources of water				
Lake				
No	281, 34	12.09 (8.29–15.91)	281, 6	2.14 (0.45–3.83)
Yes	36, 6	16.67 (4.49–28.84)		
Stream				
No	251, 28	11.16 (7.26–15.05)	251, 6	2.39 (0.05–4.28)
Yes	66, 12	18.18 (8.88–27.49)		
Pond				
No	235, 25	10.64 (6.69–14.58)	235, 4	1.70 (0.08–3.36)
Yes	82, 15	18.29 (9.92–26.66)	82, 2	2.44 (−0.90–5.78)
Developed well				
No	285, 33	11.58 (7.86–15.29)	285, 6	2.11 (0.44–3.77)
Yes	32, 7	21.875 (7.55–36.19)	32, 0	
Undeveloped well				
No	298, 37	12.42 (8.67–16.16)	298, 6	2.01 (0.42–3.61)
Yes	19, 3	15.79 (−0.61–32.19)		
Borehole				
No	242, 35	14.46 (10.03–18.89)	242, 5	2.07 (0.27–3.86)
Yes	75, 5	6.67 (1.02–12.31)	75, 1	1.33 (−1.26–3.93)
Piped tap				
No	76, 7	9.21 (2.71–15.71)	76, 1	1.32 (−1.25–3.88)
Yes	241, 33	13.69 (9.35–18.03)	241, 5	2.075 (0.28–3.87)

**Table 5:** Risk factors for fascioliasis in livestock population.

Variable	*Fasciola*-Positive (%)	cOR (95% CI) *	*p*-Value	aOR (95% CI) *	*p*-Value
Sex					
Male	31 (17.4)				
Female	32 (25.8)	1.65 (0.94–2.88)	0.079	2.17 (1.18–3.99)	0.013
Age (years)					
<2.5	15 (18.5)				
2.5–8	51 (22.3)	1.26 (0.66–2.39)	0.479		
Species					
Cattle	63 (25.0)	Ref			
Goat	1 (2.6)	0.08 (0.01–0.59)	0.013	0.05 (0.01–0.38)	0.004
Sheep	2 (10.5)	0.35 (0.08–1.57)	0.171	0.26 (0.06–1.22)	0.087
Region					
Arusha	10 (9.8)	Ref			
Kilimanjaro	30 (28.0)	3.58 (1.64–7.79)	0.001	5.37 (2.34–12.30)	0.000
Manyara	26 (25.7)	3.19 (1.45–7.03)	0.004	4.19 (1.84–9.56)	0.001
Source					
Abattoir	33 (20.9)				
Farm	33 (21.7)	1.05 (0.61–1.81)	0.859		

* cOR: Crude Odds Ratio; aOR: Adjusted Odds Ratio; CI: Confidence Interval.

**Table 6:** Risk factors for *Schistosoma* infection in human population.

Variable	*Schistosoma*-Positive *n* (%)	cOR (95% CI) *	*p*-Value	aOR (95% CI) *	*p*-Value
Gender					
Female	19 (10.5)	Ref			
Male	21 (15.4)	1.56 (0.80–3.03)	0.192		
Age (years)					
<18	12 (14.6)	Ref			
18–35	14 (10.5)	0.69 (0.30–1.57)	0.371	0.66 (0.32–1.37)	0.271
36–55	8 (14.8)	1.01 (0.38–2.67)	0.977		
Above 55	6 (12.5)	0.83 (0.29–2.39)	0.734		
Region					
Arusha	6 (5.8)	Ref			
Kilimanjaro	21 (18.4)	3.65 (1.41–9.45)	0.008	2.33 (1.15–4.71)	0.019
Manyara	13 (13.0)	2.41 (0.88–6.63)	0.087		
Education level					
No formal education	5 (15.6)	Ref			
Primary level	20 (16.7)	1.08 (0.37–3.14)	0.888		
Secondary level	12 (9.9)	0.59 (0.19–1.83)	0.365		
Tertiary level	3 (6.8)	0.39 (0.09–1.79)	0.229		
Economic activities					
Crop growing					
No	27 (11.5)	Ref			
Yes	13 (15.7)	1.42 (0.69–2.91)	0.333		
Fishing					
No	36 (12)	Ref			
Yes	4 (22.2)	2.09 (0.65–6.69)	0.216	2.81 (0.75–10.55)	0.126
Fish farming					
No	38 (12.2)	Ref			
Yes	2 (33.3)	3.59 (0.64–20.28)	0.148		
Animal husbandry					
No	27 (10.2)	Ref			
Yes	13 (25.5)	3.03 (1.48–6.38)	0.004	4.21 (1.81–9.80)	0.001
Business					
No	28 (12.1)	Ref			
Yes	12 (14.0)	1.17(0.57–2.43)	0.662		
Activities involving water contact					
Swimming					
No	34 (12.2)	Ref			
Yes	6 (15.4)	1.30 (0.51–3.34)	0.579		
Drinking					
No	4 (7.0)	Ref			
Yes	36 (13.8)	2.13 (0.73–6.24)	0.168		
Toilet					
No	2 (28.6)	Ref			
Yes	38 (12.3)	0.35 (0.06–1.86)	0.218		
Irrigation					
No	28 (11.9)	Ref			
Yes	12 (14.6)	1.27 (0.61–2.62)	0.524		
Playing (children)					
No	38 (12.8)	Ref			
Yes	2 (10.0)	0.76 (0.17–3.39)	0.716		
Bathing					
No	13 (13.0)	Ref			
Yes	27 (12.4)	0.95 (0.47–1.93)	0.890		
Fishing					
No	35 (12.9)	Ref			
Yes	5 (11.1)	0.85 (0.31–2.28)	0.743		
Providing drinking water to animals					
No	30 (11.9)	Ref			
Yes	10 (15.6)	1.38 (0.63–2.99)	0.419		
Common sources of water					
Lake					
No	34 (12.1)	Ref			
Yes	6 (16.7)	1.45 (0.56–3.74)	0.439		
Stream					
No	28 (11.2)	Ref			
Yes	12 (18.2)	1.77 (0.84–3.70)	0.130		
Pond					
No	25 (10.6)	Ref			
Yes	15 (18.3)	1.88 (0.94–3.77)	0.076	1.93 (0.93–4.03)	0.079
Developed well					
No	33 (11.6)	Ref			
Yes	7 (21.9)	2.14 (0.86–5.33)	0.103		
Undeveloped well					
No	37 (12.4)	Ref			
Yes	3 (15.8)	1.32 (0.37–4.76)	0.669		
Borehole					
No	35 (14.5)	Ref			
Yes	5 (6.7)	0.42 (0.16–1.12)	0.083	0.33 (0.11–0.97)	0.044
Piped tap					
No	7 (9.2)	Ref			
Yes	33 (13.7)	1.56 (0.66–3.69)	0.308		

* cOR: Crude Odds Ratio; aOR: Adjusted Odds Ratio; CI: Confidence Interval.

**Table 7:** Risk factors for *Fasciola* infection in human population.

Variable	*Fasciola*-Positive *n* (%)	cOR (95% CI) *	*p*-Value	aOR (95% CI) *	*p*-Value
Gender					
Female	5 (2.8)	Ref			
Male	1 (0.7)	0.26 (0.03–2.26)	0.222		
Age (years)					
<18	1 (1.2)	Ref			
18–35	1 (0.8)	0.61 (0.04–9.95)	0.731		
36–55	3 (5.6)	4.76 (0.48–47.06)	0.181	7.66 (1.36–43.23)	0.021
Above 55	1 (2.1)	1.72 (0.10–28.19)	0.703		
Crop growing					
No	3 (1.3)	Ref			
Yes	3 (3.6)	2.89 (0.57–14.59)	0.200		
Activities involving water contact					
Swimming					
No	5 (1.8)	Ref			
Yes	1 (2.6)	1.44 (0.16–12.63)	0.744		
Drinking	1 (1.7)				
No	1 (1.7)	Ref			
Yes	5 (1.9)	1.10 (0.12–9.58)	0.933		
Irrigation					
No	4 (1.7)	Ref			
Yes	2 (2.4)	1.44 (0.26–8.03)	0.675		
Fishing					
No	5 (1.8)	Ref			
Yes	1 (2.2)	1.21 (0.14–10.64)	0.861		
Providing drinking water to animals					
No	4 (1.6)	Ref			
Yes	2 (3.1)	2.01 (0.36–11.21)	0.427		
Playing (children)					
No	5 (1.7)	Ref			
Yes	1 (5)	3.07 (0.34–27.65)	0.316		
Common sources of water					
Pond					
No	4 (1.7)	Ref			
Yes	2 (2.4)	1.44 (0.25–8.03)	0.675	4.32 (0.60–30.94)	0.145
Borehole					
No	5 (2.1)	Ref			
Yes	1 (1.3)	0.64 (0.07–5.57)	0.686		
Piped tap					
No	1 (1.3)	Ref			
Yes	5 (2.1)	1.59 (0.18–13.81)	0.675		
Consumption of vegetables					
Cabbage					
No	5 (3.3)	Ref			
Yes	1 (0.6)	0.17 (0.02–1.51)	0.113	0.08 (0.01–0.89)	0.04
Spinach					
No	1 (1.5)	Ref			
Yes	5 (1.9)	1.32 (0.15–11.51)	0.801		
Chinese spinach					
No	3 (2.4)	Ref			
Yes	3 (1.6)	0.64 (0.13–3.25)	0.596		
Bitter okra					
No	3 (1.4)	Ref			
Yes	3 (3.0)	2.21 (0.44–11.13)	0.338		

* cOR: Crude Odds Ratio; aOR: Adjusted Odds Ratio; CI: Confidence Interval.

## Data Availability

The original contributions presented in this study are included in the article. Further inquiries can be directed to the corresponding author.
